# Ayurveda Treatments for Insomnia: A Narrative Review

**DOI:** 10.3390/jcm15176750

**Published:** 2026-08-31

**Authors:** Martina Vendrame, Victor Chai

**Affiliations:** Jefferson Sleep Disorders Center, Farber Institute for Neuroscience, Thomas Jefferson University, Philadelphia, PA 19107, USA

**Keywords:** insomnia, sleep architecture, *Withania somnifera*, Shirodhara, GABAergic Pathway, cortisol, Complementary and Alternative Medicine (CAM)

## Abstract

**Background:** Sleep disorders, particularly chronic insomnia (*Nidranasha*), represent an escalating global public health challenge associated with extensive neuropsychological and metabolic morbidities. While conventional pharmacotherapy provides immediate symptomatic relief, concerns regarding dependency, tolerance, and altered sleep architecture necessitate the evaluation of evidence-based complementary interventions. Ayurveda offers a comprehensive multi-modality framework for sleep health through internal adaptogenic herbs (*Abhyantar Chikitsa*), external oil-based therapies (*Bahir Parimarjana*), and structured lifestyle regimens (*Dinacharya/Ratricharya*). This narrative literature review evaluates the current clinical and mechanistic evidence supporting Ayurvedic interventions for sleep disorders. **Methods:** A focused narrative review was conducted across PubMed; Scopus; Cochrane Central Register of Controlled Trials (CENTRAL); Google Scholar; and Ayurveda, Yoga and Naturopathy, Unani, Siddha and Homoeopathy (AYUSH) Research Portal for literature published between 05/01/2016 and 05/01/2026. Randomized trials, open-label and single-arm clinical studies, mechanistic and phytochemical work, and toxicological and herb–drug interaction studies were eligible. **Results:** Standardized *Withania somnifera* root extract is supported by the most rigorous data, including double-blind placebo-controlled trials with polysomnographic or actigraphic corroboration reporting reductions in sleep-onset latency and improvements in sleep efficiency. Evidence for *Bacopa monnieri* and *Valeriana wallichii* is more limited and derives largely from studies in which sleep was a secondary outcome. External therapies, notably *Shirodhara* and *Abhyanga*, are associated with favorable autonomic and electroencephalographic changes, but the available trials are small, short, and difficult to blind, and non-specific effects of warmth, touch, and therapist attention cannot be separated from any specific treatment effect. Mechanistic work implicates modulation of gamma-aminobutyric acid (GABA) signaling, hypothalamic–pituitary–adrenal signaling, and parasympathetic activation, but these findings are hypothesis-generating and derive largely from in vitro and animal models at concentrations of uncertain human relevance. **Conclusions:** Ayurvedic interventions show preliminary signals of benefit in insomnia, most consistently for standardized *Withania somnifera*, but overall certainty remains low because of risk of bias, indirect evidence, poor standardization, and an absence of long-term safety data. Current evidence supports further rigorous investigation and carefully monitored adjunctive use in selected patients rather than general integration into routine care or substitution for established treatment.

## 1. Introduction

Sleep is a fundamental physiological process vital for survival, cellular homeostatic repair, neural plasticity, and cognitive consolidation. Globally, sleep disorders affect roughly 30% to 45% of the adult population [1,2]. Chronic sleep deprivation, sleep fragmentation, and primary insomnia are strongly linked to elevated risks of cardiovascular disease, metabolic syndrome, clinical depression, and general immune dysfunction [1,3,4]. Conventional clinical management relies heavily on gamma-aminobutyric acid (GABA) receptor agonists (such as benzodiazepines and “Z-drugs”), dual orexin receptor antagonists, and melatonin receptor agonists [2,3]. While initially effective, prolonged use of standard sedatives is frequently limited by adverse event profiles, including daytime somnolence, cognitive impairment, rebound insomnia, and psychological dependency [3]. Consequently, identifying safe, non-habit-forming therapeutic modalities remains a priority in contemporary clinical sleep medicine.

Ayurveda, an ancient traditional system of medicine originating in South Asia, categorizes sleep (Nidra) alongside food (Ahara) and controlled vitality (Brahmacharya) as one of the Trayopasthambha—the three structural pillars essential for sustaining biological life [5,6]. Within this framework, physiological sleep is driven by the natural cyclical shift where the heavy, stabilizing elements of *Kapha dosha* and psychological Tamas (inertia) pacify the kinetic, hyperactive elements of Vata and Pitta [5,7]. Pathological sleep disruption, historically termed Nidranasha or Anidra, is primarily categorized as a Vata–Pitta psychophysiological imbalance [5]. Hyperactive Vata manifests as cognitive racing, high systemic anxiety, and prolonged sleep-onset latency; aggravated Pitta typically presents as nocturnal hyperarousal, night sweats, and frequent early-morning awakenings [8].

Over the past two decades, experimental biology and clinical trials have increasingly sought to evaluate these classical concepts through a rigorous biomedical lens. A growing body of scientific literature suggest that several Ayurvedic therapies possess distinct neuropharmacological properties, directly modulating neuroendocrine pathways, circadian rhythms, and sleep architecture [9,10,11].

## 2. Materials and Methods

### 2.1. Study Design and Review Objective

This review was designed as a narrative review of the clinical and mechanistic evidence supporting Ayurvedic interventions for sleep disorders. This article aims to integrate information from randomized controlled trials (RCTs), open-label clinical series, autonomic and electrophysiological studies, receptor-level pharmacological experiments, in vivo animal models, phytochemical standardization analyses, and classical Ayurvedic textual sources. The objective was not to enumerate every publication containing the terms “Ayurveda” and “sleep”, but to interpret the evidence and limitations of specific Ayurvedic modalities such as internal adaptogenic botanicals, external oil-based therapies, and structured circadian regimens and determine if they produce measurable changes in sleep onset latency, sleep continuity, sleep architecture, and the neuroendocrine substrates of hyperarousal.

The review examined monographic and polyherbal formulations (*Abhyantar Chikitsa*), specialized external therapies (*Bahir Parimarjana*), and lifestyle protocols (*Dinacharya*/*Ratricharya*), highlighting their molecular mechanisms, clinical efficacy, and existing limitations within current research. Mechanistic, phytochemical, toxicological, and herb–drug interaction studies were assessed to address translational barriers to clinical adoption. Because this article is designed as a narrative review rather than systematic review with meta-analysis, no pooled effect estimate was calculated, no protocol was registered, and no Preferred Reporting Items for Systematic Reviews and Meta-Analyses (PRISMA) flow diagram was produced. The design, structure, and reporting of this review were guided by the Scale for the Assessment of Narrative Review Articles (SANRA) framework.

### 2.2. Literature Sources and Search Strategy

To map the scientific landscape of Ayurvedic sleep interventions, a targeted search was conducted across PubMed; Scopus; Cochrane Central Register of Controlled Trials (CENTRAL); Google Scholar; and Ayurveda, Yoga and Naturopathy, Unani, Siddha and Homoeopathy (AYUSH) Research Portal. The searches were executed between 1 January 2026 and 6 January 2026 for articles published between 5 January 2016 and 5 January 2026.

An iterative search approach was utilized to synthesize the literature for this narrative review. Initial baseline queries combined general Ayurvedic and sleep concepts and were followed by intervention-specific searches (*Withania somnifera*, *Bacopa monnieri*, *Valeriana wallichii*, polyherbal and *Ghrita*-based formulations, *Shirodhara*, *Abhyanga*, *Padabhyanga*, *Pranayama*), by mechanism-specific searches (GABAergic modulation, hypothalamic–pituitary–adrenal (HPA) axis and cortisol dynamics, heart rate variability, brain-derived neurotrophic factor (BDNF) expression, sleep architecture), and by translational searches addressing phytochemical standardization, heavy-metal contamination, and herb–drug interactions. In addition, forward citation searching was performed to capture emerging data and conflicting findings.

### 2.3. Search Terms

Search terms were combined using Boolean operators and adapted to the syntax and indexing vocabulary of each database. In PubMed, Medical Subject Headings (MeSH) terms were combined with title/abstract free-text terms and truncation. The core strategy was:

Intervention concept: Ayurved*[tiab] OR “Medicine, Ayurvedic”[MeSH] OR “Withania somnifera”[tiab] OR Ashwagandha[tiab] OR withanolide*[tiab] OR “Bacopa monnieri”[tiab] OR Brahmi[tiab] OR bacoside*[tiab] OR Valeriana[tiab] OR Tagara[tiab] OR “valerenic acid”[tiab] OR Shirodhara[tiab] OR Abhyanga[tiab] OR Padabhyanga[tiab] OR Rasayana*[tiab] OR Medhya[tiab] OR Panchakarma[tiab] OR Pranayama[tiab] OR “Breathing Exercises”[MeSH];

Outcome concept: “Sleep Initiation and Maintenance Disorders”[MeSH] OR “Sleep Wake Disorders”[MeSH] OR “Sleep”[MeSH] OR insomnia[tiab] OR Nidranasha[tiab] OR Anidra[tiab] OR sleep*[tiab] OR “Polysomnography”[MeSH] OR polysomnograph*[tiab] OR actigraph*[tiab] OR “Pittsburgh Sleep Quality Index”[tiab] OR “Insomnia Severity Index”[tiab];

These two concepts were combined with AND. Three supplementary searches were run in parallel and combined with OR: (i) insomnia epidemiology, nosology, and conventional pharmacotherapy, using insomnia terms combined with epidemiolog*, prevalence, burden, guideline*, hyperarousal, “Benzodiazepines”[MeSH], z-drug*, zolpidem, and “orexin receptor antagonist*”; (ii) safety and quality control, combining Ayurvedic and herbal medicine terms with “Metals, Heavy”[MeSH], lead, mercury, arsenic, contaminat*, adulterat*, standardi*, chromatograph*, “Herb-Drug Interactions”[MeSH], “Cytochrome P-450 Enzyme System”[MeSH], and pharmacovigilance; and (iii) mechanistic pathways, combining the named botanicals and external therapies with “Receptors, GABA-A”[MeSH], GABA*, “Hydrocortisone”[MeSH], cortisol, “HPA axis”, “Brain-Derived Neurotrophic Factor”[MeSH], BDNF, “heart rate variability”, parasympathetic, and electroencephalograph*.

### 2.4. Eligibility Criteria

The eligibility criteria included randomized controlled trials evaluating Ayurvedic internal or external interventions for sleep quality or clinically defined insomnia; prospective open-label and single-arm clinical studies employing validated subjective instruments such as the Pittsburgh Sleep Quality Index (PSQI) and Insomnia Severity Index (ISI) or objective biometric measures (polysomnography, actigraphy, heart rate variability, salivary or serum cortisol); systematic reviews and meta-analyses of the above; in vivo and in vitro mechanistic studies elucidating the neurochemical, neuroendocrine, or autonomic pathways of specific Ayurvedic herbs, formulations, or procedures; analytical and phytochemical studies addressing standardization of marker compounds; and toxicological, pharmacovigilance, and herb–drug interaction studies relevant to clinical safety. Classical Ayurvedic texts in published English translation were included solely to establish traditional diagnostic and therapeutic framing.

Studies not eligible for consideration included publications in languages other than English without an available validated English translation; grey literature, preprints, and non-peer-reviewed commentary; conference abstracts lacking extractable outcome data; and studies in which the intervention was not identifiable as an Ayurvedic modality.

Articles that were excluded included trials lacking clearly defined diagnostic criteria for the sleep disturbance under study; studies in which the Ayurvedic intervention could not be separated from co-administered pharmacological or behavioral treatments; and studies reporting no sleep-related, neuroendocrine, autonomic, or safety outcome.

### 2.5. Evidence Selection, Data Organization, and Narrative Synthesis

Given the narrative scope of this review, study selection was guided by clinical applicability, study design rigor, and conceptual contribution rather than a systematic synthesis. Methodological priority was assigned to double-blind, placebo-controlled randomized controlled trials (RCTs), investigations employing objective polysomnographic or actigraphic measures, systematic reviews, meta-analyses, and studies evaluating defined neurobiological mechanisms. Rather than utilizing standardized risk-of-bias scoring tools or formal certainty frameworks such as the Grading of Recommendations, Assessment, Development and Evaluations (GRADE) approach, study quality was evaluated qualitatively. Single-reviewer selection and extraction were conducted, which can increase the risk of error and selection bias.

## 3. Results

### 3.1. Internal Ayurvedic Pharmacotherapy (Abhyantar Chikitsa)

Internal interventions utilize specific single herbs (*Eka-Herbs*) or synergistic polyherbal compounds classified as *Medhya Rasayanas* (nootropic, neuro-rejuvenating therapies). These agents work primarily by modulating neurotransmitter cascades and mitigating oxidative stress within the central nervous system [12].

### 3.2. Withania Somnifera (Ashwagandha)

*Withania somnifera* is the most extensively researched Ayurvedic botanical for sleep health [9,10]. The bioactive constituents responsible for its therapeutic properties include a class of steroidal lactones known as withanolides (predominantly withaferin A and withanolide D), alongside high concentrations of triethylene glycol (TEG) [12,13].

Mechanistic Basis

*Rodent models* demonstrate that *Withania somnifera* extracts potentially increase GABA content in the brain and exhibit GABA-A receptor agonism while upregulating mRNA and protein expression levels of GABA-A, GABA-B, and 5-hydroxytryptamine 1A (5-HT1A) receptors [14]. Electrophysiological studies, however, may suggest that increased withanolide or withaferin A content does not induce sleep or activate GABA-A receptors [14]. Furthermore, TEG has been isolated as a potent sleep-inducing component that acts independently to clear hyperarousal pathways and promote non-rapid eye movement (NREM) sleep [13]. Ashwagandha also acts as a potent adaptogen by down-regulating the expression of c-Fos in the paraventricular nucleus of the hypothalamus, dampening the cascade of adrenocorticotropic hormone (ACTH) and reducing systemic circulating cortisol [15]. These proposed mechanisms, however, have not been tested in human pharmacokinetics.

Clinical Efficacy

Numerous high-quality, double-blind, placebo-controlled RCTs have evaluated Ashwagandha root extract in both healthy individuals and patients with chronic insomnia [9,15]. A multi-center 8-week RCT evaluating a standardized aqueous extract (300 mg twice daily) noted a significant reduction in Sleep-Onset Latency (SOL) by an average of 15.4 min (*p* < 0.001) compared to the placebo group [9]. Sleep efficiency (SE) scores improved by over 9%, accompanied by substantial reductions on the Hamilton Anxiety Rating Scale (HAM-A) [9,15]. Polysomnographic validations confirm that Ashwagandha selectively increases the duration of slow-wave sleep (Stage N3) and stabilizes sleep architecture without inducing morning sluggishness or withdrawal rebound [10].

### 3.3. Bacopa Monnieri (Brahmi)

*Bacopa monnieri* is a classical *Medhya* botanical traditionally utilized to calm hyperactive mental states and improve sleep quality disrupted by emotional stress or cognitive fatigue [8,16].

Mechanistic Basis

The neuroprotective and anxiolytic efficacy of *Bacopa monnieri* is driven by its dammarane-type triterpenoid saponins, known as bacosides (bacoside A and B) [16]. Saponins from *B. monnieri* modulate central monoaminergic transmission, concurrently upgrading serotonin synthesis via the upregulation of tryptophan hydroxylase-2 (TPH2) while facilitating the expression of vesicular glutamate transporters to control excitotoxicity [16]. Additionally, Brahmi enhances neuronal expression of brain-derived neurotrophic factor (BDNF) within the hippocampus, mitigating the neurostructural damage associated with chronic, insomnia-induced sleep deprivation [16].

Clinical Efficacy

In clinical settings, Brahmi is rarely used as a standalone heavy sedative; rather, it serves to optimize sleep continuity and reduce mid-night awakenings (*Khandita Nidra*) [8]. Clinical trials using standardized *B. monnieri* extracts (300–450 mg/day) show a major reduction in stress-induced sleep fragmentation [17]. Patients report enhanced cognitive clarity, memory recall, and lower daytime fatigue scores upon waking, illustrating its capacity to balance Pitta hyper-reactivity [8].

### 3.4. Valeriana Wallichii (Tagara)

Known colloquially as Indian Valerian, *Valeriana wallichii* is classified within Ayurveda as a potent *Nidrajanana* (sleep-inducing) drug used to address acute Vata instability [18].

Mechanistic Basis

Tagara contains iridoid esters (valepotriates) and valerenic acid [18,19]. Valerenic acid binds directly to the beta-subunit of the GABA-A receptor, stimulating a profound hyperpolarization of the postsynaptic membrane [19]. Concurrently, it inhibits the enzyme GABA transaminase, preventing the metabolic breakdown of synaptic GABA and maintaining elevated neurotransmitter levels within the synaptic cleft [19].

Clinical Efficacy

Clinical studies indicate that *V. wallichii* extract (500–1000 mg of hydroalcoholic extract at bedtime) acts effectively as a natural sleep inducer [18]. In comparative clinical trials, Tagara significantly reduced SOL and improved overall sleep quality scores on the PSQI [18]. While its sedative profile is more pronounced than that of Ashwagandha, long-term safety studies show a lower incidence of daytime motor incoordination compared to conventional benzodiazepines [18,19].

### 3.5. Synergistic Polyherbal Formulations

Ayurvedic clinical practice relies heavily on polyherbal matrix engineering, combining multiple plants to produce synergistic effects while minimizing side effects [20]. Key formulations include:

- Brahmi Ghrita: A lipid-based preparation where *Bacopa monnieri*, *Convolvulus pluricaulis* (Shankhpushpi), and *Acorus calamus* (Vacha) are infused into clarified butter (ghee).
- Saraswatarishta: A naturally fermented biotransformed liquid formulation containing *Bacopa monnieri*, and *Asparagus racemosus* (Shatavari) [21]. The natural fermentation process yields plant-derived polyphenols and short-chain organic acids that optimize gastrointestinal assimilation, correcting the *Agni* (digestive fire) alterations that frequently accompany chronic sleep disorders [21].

## 4. External and Physical Ayurvedic Interventions (Bahir Parimarjana)

External treatments bypass gastrointestinal digestion, leveraging the extensive somatosensory network of the skin and cranial nerves to induce systemic physiological changes [11,22].

### 4.1. Shirodhara (Forehead Oil Therapy)

*Shirodhara* involves pouring a continuous, warm stream of medicated oil (such as *Ksheerabala Taila* or *Brahmi Taila*) over the glabrous skin of the forehead, specifically targeting the glabella or *Ajna Chakra* [11,23].

Physiological Mechanism

The forehead contains a dense concentration of mechanoreceptors (Merkel discs and Meissner corpuscles) and thermoreceptors innervated by the ophthalmic branch of the trigeminal nerve [11]. The constant, gentle pressure and warmth of the oil create a rhythmic somatosensory stimulus [23].

Neuroimaging and autonomic monitoring reveal that *Shirodhara* decreases the Low-Frequency to High-Frequency (LF/HF) ratio of Heart Rate Variability (HRV), indicating a significant shift from sympathetic hyperarousal to parasympathetic dominance [11]. Electroencephalographic (EEG) readings during *Shirodhara* show an immediate increase in alpha and theta wave activity, matching the neurophysiological signatures of deep meditation and the early stages of NREM sleep [11,23,24].

Clinical Evidence

In a controlled clinical trial evaluating patients with severe primary insomnia, a course of 14 daily *Shirodhara* sessions yielded a 42% reduction in Insomnia Severity Index (ISI) scores [23,25]. Objective actographic markers validated a corresponding decrease in Wake After Sleep Onset (WASO) and a significant improvement in overall sleep efficiency [25].

### 4.2. Abhyanga and Padabhyanga (Therapeutic Massage)

*Abhyanga* refers to full-body oil massage, while *Padabhyanga* targets the feet and lower limbs using specialized oils like warm sesame or coconut oil [22,26].

Mechanistic Basis

Tactile skin stimulation during *Abhyanga* activates large-diameter myelinated A-beta nerve fibers, which down-regulates the nociceptive signaling of smaller, unmyelinated C-fibers within the spinal cord dorsal horn (the gate control theory of afferent modulation) [22]. This systemic reduction in physical tension triggers a cascade release of endogenous oxytocin and serotonin while reducing circulating epinephrine and norepinephrine [22].

Clinical Evidence

Clinical trials confirm that a structured manual foot massage significantly improves subjective sleep latency, sleep quality (PSQI), and insomnia severity. The intervention is proposed to shift the autonomic balance towards parasympathetic dominance reflected by reduced heart rates, elevated heart rate variability, and decreased LF/HF spectral ratio. [26]. Padabhyanga can be an effective, low-cost home intervention for mild-to-moderate sleep disturbances [26].

## 5. Circadian Medicine and Lifestyle Protocols (Dinacharya/Ratricharya)

Ayurvedic sleep medicine emphasizes synchronization with natural environmental cycles, preceding modern chronobiology by millennia [27]. The daily routine (*Dinacharya*) and nightly routine (*Ratricharya*) mandate structured lifestyle adjustments to maintain circadian health [5,27].

- Solar Synchronization: Ayurveda advocates waking during the *Brahma Muhurta* (approximately 48 min before sunrise), a period when environmental Vata is dominant, which helps optimize cortisol awakening responses (CAR).
- Dietary Chrononutrition (*Ahara Timing*): The primary meal must be consumed at midday when solar energy and digestive capacity (Agni) peak [5]. Dinner must be light, balancing Vata–Pitta, and completed at least 2.5 to 3 h before sleep [7]. This prevents overnight gastroesophageal reflux and avoids nocturnal insulin spikes that can disrupt growth hormone and melatonin secretion patterns.
- Pranayama and Mindfulness: Practices such as *Nadi Shodhana* (alternate nostril breathing) and *Bhramari* (bumblebee breath) activate the vagus nerve [28]. This breathwork increases respiratory sinus arrhythmia, lowering central sympathetic tone to facilitate a smooth transition into physiological sleep [28].

## 6. Discussion

### 6.1. Classical Ayurvedic Framework vs. Contemporary Sleep Pathophysiology

To integrate traditional therapies into evidence-based clinical protocols, it is necessary to map classical concepts to modern biomedical equivalents. The Ayurvedic etiology of *Nidranasha* aligns closely with the hyperarousal model of chronic insomnia [29].

According to Ayurvedic texts (such as the *Charaka Samhita* and *Sushruta Samhita*), *Nidranasha* arises when mental stress (Chinta), physical trauma (Abhighata), or emaciating dietary habits deplete the body’s essential vitality (Ojas) [7]. This depletion permits Vata (characterized by dryness, mobility, and coldness) to infiltrate the sensory and cognitive pathways (*Manovaha Srotas*), keeping the nervous system in a state of hyper-vigilance [7].

In contemporary neurobiology, this state is recognized as a chronic dysregulation of the hypothalamic–pituitary–adrenal (HPA) axis, causing elevated nocturnal cortisol levels, increased sympathetic nervous system (SNS) tone, and a corresponding deficit in central gamma-aminobutyric acid (GABA) signaling [15]. Table 1 details this direct clinical mapping.

### 6.2. Comparative Analysis: Ayurveda vs. Conventional Sleep Medicine

To contextualize Ayurveda’s role in modern clinical settings, it is helpful to contrast its clinical profiles directly with standard allopathic pharmaceutical options (Table 2). The comparison with cognitive behavioral therapy for insomnia (CBT-I) is also instructive because it is the guideline-recommended first-line treatment for chronic insomnia [3]. A recent systematic review and meta-analysis of 53 randomised controlled trials (n = 13,608) found that CBT-I remained superior to control conditions in reducing depressive symptoms at 3 months (k = 29, d = −0.35, 95% Confidence Interval (CI) −0.49 to −0.21), 6 months (k = 29, d = −0.32, 95% CI −0.59 to −0.05), and 12 months (k = 10, d = −0.25, 95% CI −0.39 to −0.12) post-treatment, indicating that benefit is maintained across the year following the intervention and extends beyond sleep disorders [31].

## 7. Safety, Toxicology, and Quality Control Challenges

Despite promising clinical evidence, integrating Ayurvedic sleep treatments into mainstream medicine requires addressing safety, toxicology, and quality control [20,33].

### 7.1. Heavy Metal Contamination and Adulteration

A primary challenge in global Ayurvedic distribution is the potential presence of heavy metals (such as lead, mercury, and arsenic) [33]. While specialized metallic formulations (*Rasa Shastra*) utilize complex purifications (*Shodhana*) to alter metal bioavailability, accidental contamination of standard herbal products can occur due to poor agricultural soil quality or inadequate manufacturing oversight [20,33]. To meet international safety guidelines such as those from the U.S. Food and Drug Administration (FDA) and European Medicines Agency (EMA), all herbal materials must undergo strict validation via Inductively Coupled Plasma Mass Spectrometry (ICP-MS) to ensure heavy metal counts remain safely below permissible daily exposure limits [33,35].

### 7.2. Standardization of Polyherbal Formulations

Because botanical profiles vary based on geographic location, climate, and harvest timing, raw herbs can exhibit significant batch-to-batch variations in their active chemical constituents [20,34]. For instance, the concentration of withanolides in *Withania somnifera* can fluctuate markedly between crops [34]. Manufacturers must use High-Performance Thin-Layer Chromatography (HPTLC) and High-Performance Liquid Chromatography (HPLC) to confirm standardized concentrations of key chemical markers (e.g., asserting a minimum of 5% withanolides for Ashwagandha or 20% bacosides for Brahmi extracts) across production cycles [20,34].

### 7.3. Herb–Drug Interactions

As more patients combine complementary therapies with conventional medications, understanding herb–drug interactions is critical [32]:

- GABAergic Synergism: Combining *Valeriana wallichii* (Tagara) or Ashwagandha with alprazolam, zolpidem, or barbiturates can cause excessive central nervous system depression and increased daytime drowsiness due to synergistic action at the GABA receptor site [18,32].
- Cytochrome P450 Modulation: Active constituents in *Bacopa monnieri* have been shown to moderately inhibit CYP2C9 and CYP3A4 enzymes in the liver [16,32]. This inhibition can elevate serum concentrations of concurrently administered pharmaceuticals, including certain anticoagulants, statins, and anticonvulsants, requiring careful monitoring by clinicians [32].

## 8. Methodological Limitations in Existing Ayurvedic Literature

While current research supports the therapeutic value of Ayurvedic sleep interventions, a critical analysis reveals several methodological limitations in the existing literature [20,36]:

1. Small Sample Sizes: Many published clinical trials operate with small cohorts (frequently n < 50), which limits the statistical power needed to generalize findings across broader, ethnically diverse patient populations [9,18,36].
2. Lack of Objective Outcome Measures: A significant proportion of Ayurvedic trials rely primarily on subjective sleep logs or self-reported questionnaires (like the PSQI or ISI) [18,25]. While patient-reported outcomes are important, studies frequently lack validation from objective biometrics, such as overnight polysomnography or multi-day actigraphy [10,36].
3. Blinding Challenges with External Therapies: Double-blinding presents an inherent logistical challenge in trials involving physical interventions like *Shirodhara* or *Abhyanga* [11,26]. Therapists and patients can easily identify the active treatment, introducing potential performance and detection biases that can inflate observed therapeutic effects [36].
4. Heterogeneity in Treatment Formulations: Variations in vehicle substances (such as using different base oils or decoctions) and differing lengths of treatment cycles across studies make it difficult to perform rigorous meta-analyses or establish standardized clinical protocols [20,36].

A critical overarching limitation in evaluating the clinical efficacy of Ayurvedic sleep interventions is that RCTs constitute only a small fraction of the total published literature in this field. The overarching conclusions synthesized across contemporary literature reviews, including those presented in this paper, are broadly derived from an aggregate analysis of all published evidence, which relies heavily on non-randomized prospective cohorts, open-label clinical series, uncontrolled pilot studies, and preclinical models. Differentiating between high-tier RCT data and the broader body of published literature is vital because non-randomized and unblinded study designs are exceptionally vulnerable to non-specific therapeutic variables, regression to the mean, expectation bias, and, most prominently, a powerful placebo response.

Within clinical sleep medicine, the placebo effect can be robust. This vulnerability to placebo effects is further amplified within the context of Ayurvedic therapeutics due to the deeply tactile, sensory, and ritualistic components inherent to treatments such as Shirodhara (forehead oil pouring), Abhyanga (full-body massage), and warm herbal decoctions. These experiential therapies inherently cultivate deep somatic relaxation, psychological reassurance, and immediate parasympathetic shifts that can occur entirely independent of any specific active pharmacological compound or targeted mechanism. Consequently, clinical trials lacking rigorous placebo controls or active sham arms risk substantially overestimating therapeutic efficacy by misattributing non-specific, expectation-mediated improvements to inherent drug or treatment properties.

If the analytical scope of this review were strictly restricted to evidence derived exclusively from rigorous, double-blind, placebo-controlled RCTs, the primary conclusions regarding Ayurvedic sleep interventions would inevitably change and become far more restricted. Under such restrictive criteria, many widely used polyherbal matrices, complex biotransformed preparations, specialized external procedures, and chronotherapeutic lifestyle protocols would currently lack sufficient empirical evidence to support definitive, evidence-based clinical recommendations. Instead, proven efficacy would be confined to a narrow subset of standardized single botanicals, most notably Withania somnifera (Ashwagandha), that have successfully demonstrated objective improvements in sleep architecture across double-blind RCTs validated with polysomnography or actigraphy. Transparently recognizing that current conclusions reflect the totality of published evidence rather than an exclusive corpus of RCT data does not diminish the clinical promise of traditional Ayurvedic modalities, but it underscores the imperative for methodological evolution. To establish a universally accepted role within contemporary sleep medicine, future research must prioritize large-scale, double-blind RCT architectures incorporating active sham controls and standardized matrix preparations capable of isolating true physiological efficacy from non-specific contextual placebo effects.

## 9. Future Research Directions

To advance the integration of Ayurvedic therapies into mainstream sleep medicine, future research must prioritize large-scale, multi-center, double-blind RCTs that utilize objective biometric end-points, including polysomnographic sleep architecture analysis, actigraphic movement monitoring, and longitudinal salivary cortisol profiles [10,25,36]. Researchers should also focus on exploring the preventive and therapeutic potential of these modalities for specific sleep phenotypes, such as shift-work sleep disorder, age-related sleep fragmentation, and insomnia secondary to post-traumatic stress disorder (PTSD) [1,37].

Despite the mounting body of empirical, neurochemical, and mechanistic evidence supporting the therapeutic utility of Ayurvedic modalities for sleep disorders, there has been a noticeable fading of interest within the broader academic sleep medicine community. While initial exploratory trials and mechanistic discoveries sparked substantial intrigue regarding adaptogenic botanical pathways and parasympathetic somatosensory modulation, recent years have seen a palpable decline in enthusiasm. This waning engagement is reflected in a diminishing presence of Ayurvedic research at mainstream international sleep conferences, a reduction in dedicated research funding from major sleep research organizations, and a relative scarcity of high-impact publications within tier-one sleep medicine journals.

Several systemic factors contribute to this growing disconnect between expanding preliminary evidence and stagnating institutional interest. A primary driver is the fundamental paradigm friction between traditional Ayurvedic holistic frameworks and contemporary Western medical reductionism. Western sleep medicine has increasingly shifted toward highly targeted pharmacotherapy—such as dual orexin receptor antagonists—and standardized behavioral interventions like Cognitive Behavioral Therapy for Insomnia (CBT-I). In contrast, the multi-component, highly individualized nature of Ayurvedic treatment plans creates persistent methodological hurdles for standard clinical research models. The sleep research community frequently views the inability to easily isolate single active compounds from complex polyherbal formulations, alongside the logistical difficulty of constructing true double-blind sham controls for external therapies like Shirodhara, as a barrier to achieving the level of evidence required for clinical adoption.

Furthermore, persistent issues regarding product quality control, heavy metal contamination, and variable standardization metrics across international markets have contributed to an enduring skepticism among clinical sleep specialists. Without universal manufacturing oversight and reliable batch-to-batch chemical uniformity, many mainstream clinicians remain hesitant to advocate for or investigate these interventions, fearing potential herb–drug interactions or safety liabilities. Consequently, as the broader sleep community concentrates its resources on precision medicine, neuroimaging, and digital therapeutics, Ayurvedic sleep medicine risks being relegated to a niche area of interest. To reignite enthusiasm and bridge this expanding divide, future studies must directly address these institutional concerns by leveraging standardized phytotherapeutic extracts, rigorous safety profiling, and collaborative, multi-center trials designed to meet the rigorous evidence standards demanded by modern sleep science.

## 10. Conclusions

In conclusion, the current scientific literature demonstrates that Ayurvedic treatments may offer an evidence-based approach to managing sleep disorders. By combining internal adaptogenic botanicals that modulate GABAergic and HPA axis pathways with external somatosensory therapies that enhance parasympathetic tone, Ayurveda may address the underlying physiological imbalances driving chronic hyperarousal. Currently, Ayurvedic interventions show preliminary signals of benefit but remain limited because of risk of bias, indirect evidence, poor standardization, and insufficient long-term safety data. When supported by rigorous chemical standardization, transparent safety protocols, and robust clinical trial methodologies, these traditional interventions may serve as safe, effective alternatives or adjuncts within contemporary sleep medicine frameworks.

## Figures and Tables

**Table 1:** Translational Mapping of Ayurvedic and Western Sleep Pathophysiology.

Ayurvedic Diagnostic Concept	Proposed Conceptual Parallel	Primary Pathophysiological Manifestation
Vata Aggravation (*Chala/Ruksha Guna*)	Sympathetic Hyperarousal & GABAergic deficit	Prolonged Sleep-Onset Latency (SOL); high baseline anxiety; somatic tension [15].
Pitta Aggravation (*Ushna/Tikshna Guna*)	HPA-Axis Dysregulation & Pro-inflammatory status	Wake After Sleep Onset (WASO); frequent nocturnal awakenings; disrupted thermal homeostasis [8].
Tarpaka Kapha Depletion	Reduction in Slow-Wave Sleep (SWS) & Delta Power	Unrefreshing sleep; fragmented deep sleep architecture; cognitive fatigue [7,10].
Manovaha Srotas Blockage	Impaired Neuroplasticity & Glymphatic Clearance Dysfunction	Accumulation of metabolic waste (e.g., β-amyloid); chronic low-grade neuroinflammation [12,21,30].

**Table 2:** Comparative Clinical Matrix of Sleep Interventions.

Intervention Class	Principal Mechanism	Evidence Identified in This Review	Reported Effects on Sleep	Safety and Dependency	Certainty (Qualitative)
Cognitive behavioral therapy for insomnia (CBT-I)	Behavioral and cognitive modification of sleep-related arousal and conditioning	Guideline-level synthesis; recommended as first-line treatment for chronic insomnia in adults [3]	Improvement in sleep onset, maintenance, and sleep efficiency sustained after treatment ends [3]	No pharmacological adverse effects; limited by access, cost, and adherence [3]	High [3]
Benzodiazepines	Positive allosteric modulation at GABA-A receptors; non-subunit-selective [3]	Guideline-level synthesis; efficacy established for short-term use [3]	Reduces sleep-onset latency and wake after sleep onset; reduces slow-wave and REM sleep [3]	Daytime sedation, cognitive slowing, psychomotor impairment, falls; tolerance, dependence, and rebound insomnia on discontinuation. Long-term use not recommended [3]	Moderate–high for short-term efficacy; long-term benefit not established [3]
Benzodiazepine receptor agonists (Z-drugs)	Positive allosteric modulation with relative α1-subunit preference [3]	Guideline-level synthesis; efficacy established for short-term use [3]	Reduces sleep-onset latency; effects on sleep architecture less pronounced than benzodiazepines [3]	Similar class-level concerns to benzodiazepines, including dependence and complex sleep behaviors; long-term use not recommended [3]	Moderate–high for short-term efficacy; long-term benefit not established [3]
Melatonin (prolonged release) and melatonin receptor agonists	Chronobiotic action at melatonin receptor 1 and 2 (MT1/MT2) receptors [3]	Guideline-level synthesis [3]	Small effects on sleep-onset latency; not recommended for routine treatment of chronic insomnia on current evidence [3]	Favorable short-term tolerability profile [3]	Low [3]
*Withania somnifera* (Ashwagandha), standardized root extract	Proposed GABAergic and HPA-axis modulation; rodent data indicate increased brain GABA content and receptor expression, though withanolide content alone did not induce sleep [14]; human hormonal outcomes reported [15]	Double-blind, placebo-controlled RCTs in insomnia and anxiety [9]; polysomnographic evaluation [10]; placebo-controlled human study of stress and hormonal outcomes [15]. Small single-centre samples; placebo comparator only	Reduced sleep-onset latency and improved sleep efficiency vs. placebo [9]; polysomnographic changes in sleep architecture reported [10]	No serious adverse events reported in the included trials [9,15]. Hepatotoxicity, thyroid effects, and cytochrome P450 (CYP)-mediated interaction remain incompletely characterized [32]; product-level heavy-metal contamination documented for Ayurvedic preparations generally [33]; marker-compound content varies between batches [34]	Low—randomized and placebo-controlled, but small, single-centre, short-duration, no active comparator
*Bacopa monnieri* (Brahmi)	Proposed monoaminergic and neurotrophic modulation [16]	No trial with a primary sleep endpoint identified; available clinical synthesis addresses cognitive and affective outcomes [16]	Sleep outcomes secondary or not assessed	CYP2C9 and CYP3A4 inhibition reported [32]; contamination and standardization concerns as above [33,34]	Very low for sleep—indirect outcome, no sleep-primary trial identified
*Valeriana* spp.	Proposed GABAergic modulation [19]	Systematic review and meta-analysis of *Valeriana officinalis* [18]. No trial of *V. wallichii* identified; species indirectness must be stated	Subjective sleep-quality improvement reported; objective corroboration limited [18]	Guideline [3] does not recommend phytotherapeutic agents for insomnia on current evidence	Very low—species indirectness plus guideline non-recommendation
*Shirodhara*	Proposed autonomic and cortical modulation via trigeminal somatosensory stimulation	Randomized comparison of sesame oil vs. water [23]; comparative trial of three *Shirodhara* media [25]; feasibility/pilot study within an integrated yoga programme in anxiety disorder [11]	Improvement in subjective sleep measures and autonomic indices reported [11,23,25]	No serious adverse events reported; trials are short and small	Very low—pilot and small comparative designs, non-blindable; non-specific effects of warmth, touch, and therapist contact not separable
*Abhyanga*/*Padabhyanga*	Proposed parasympathetic activation via tactile stimulation [22]	Massage therapy research review [22]; programmed reflexology therapy trial reporting sleep and autonomic outcomes [26]	Improved subjective sleep quality and insomnia severity; reduced heart rate, increased HRV, reduced LF/HF ratio [26]	No serious adverse events reported	Very low—modality indirectness: reflexology is not *Padabhyanga*; must be stated explicitly

## Data Availability

No new data were created or analyzed in this study. Data sharing is not applicable to this article.
